# The Clinical Importance of the Examination of Feces1Read before the Buffalo Academy of Medicine, Section on Pathology.

**Published:** 1896-09

**Authors:** Julius Ullman

**Affiliations:** Buffalo, N. Y. 400 Franklin Street


### THE CLINICAL IMPORTANCE OF THE EXAMINATION OF FECES.[1]

By JULIUS ULLMAN, M. D., Buffalo, N. Y.

GREAT progress has been made in accurate and scientific methods of determining the secretions and excretions of the body, as related to the normal and pathological conditions. As an instance of this progress, I would refer to the discovery of the etiological rôle played by the Laveran plasmodium malariæ in the determination of malarial fever. Every clinician today examines the blood as to its behavior in the primary and secondary anemias, and the physical examination of a phthisical patient is incomplete until the tubercle bacillus can be demonstrated under the impartial eye of the microscope, as the specific organism in this disease. No one can accurately ascertain the pathogenesis of the genito-urinary tract without examination with the microscope of the gonococcus within the pus-cells in gonorrhea, or the examination of the sediment in vesical or nephritic diseases. The profession today is thankful to Ewald, Boas and Einhorn for their original work in illuminating general medicine by their chemical and microscopic examination of stomach contents.

The object of this paper is to impress upon you the necessity for a closer study of the contents of the intestinal tract. It is true that studying the feces is not the most agreeable or esthetic task, but it is not more disagreeable to accustom our fine sense of smell to this work than to the post mortem room.

The feces consist, according to Nothnagel,[1] of (1) changed or unchanged remains of the food consumed ; (2) bacteria ; (3) portions of the intestinal and gastric secretions ; (4) formed and unformed elements from the intestinal wall ; and (5) salts which are the products of digestion.

We study the dejecta macroscopically, microscopically and bacteriologically. Macroscopically we study them as to their frequency and amount, consistency and form, color, odor and reaction. The frequency and amount are both dependent upon the food stuffs ingested. In a healthy person there occur several stools daily ; in others there is catharsis every other day, and between these extremes there are all sorts of gradations. In a constipation the irregularity of evacuating the bowel is accompanied by symptoms of disease, and the food stuffs ingested are not

1. Read before the Buffalo Academy of Medicine, Section on Pathology.

proportional to the feces excreted; in diarrhea the stools are not only frequent but also liquid in character.

Rubner found that after taking 2,438 gms. of milk, 120.8 gms. of feces were excreted, and after taking 1,435 gms. of meat 81.2 gms. of feces were evacuated. Vegetables increase the amount, so that in an ordinary mixed diet Nothnagel has demonstrated that 150 to 180 gms. of fecal matter are passed in twenty-four hours. In the starving the stools are reduced to a minimum, and here it must not be forgotten that in many or all disease processes the patient is in a starving condition. Diseases which decrease the frequency and amount of the stool by the accompanying constipation are: (1) chronic enteric catarrh; (2) gastrectasis; (3) stenoses, esophageal, cardiac, pyloric or intestinal; (4) peritonitis; (5) meningitis and other affections causing intracranial pressure; (6) chronic obstipation, *per se*, which is nervous in origin, and in which the peristalsis of the colon and rectum is paralysed. This variety is encountered in tabes dorsalis. On the other hand, diseases which increase the frequency by the accompanying diarrhea are: (1) gastric catarrh; (2) chronic peritonitis; (3) tuberculosis of the intestinal tract; (4) amyloid degeneration of the intestines; (5) hepatic cirrhosis; (6) nephritis with uremia; (7) cholera, dysentery; (8) typhoid fever and other infectious diseases.

Blood and mucus can increase the volume of the stool, and after a constipation the dejecta are temporarily increased.

The normal consistency of the stool is soft, doughy and the form is familiar to all; the consistency is dependent upon the relative amount of water in the dejecta. In a general way it may be said that the longer the feces remain in the large intestine, the more solid will they be evacuated and, *vice versa*, the shorter the contact with the walls of the large intestines the more liquid will they appear.

In a high lying stenosis of the intestine the feces may be decreased in diameter and may appear long, thin, drawn out and in the shape of a lead pencil; but this need not necessarily be, for on their passage downward to the anus they may be reformed again. On the other hand, if the seat of stricture is in close proximity to the anus the dejecta will always appear in the form described above. A stool such as is mentioned need not necessarily point to a stricture due to an organic lesion, for such stools are also passed in nervous spastic conditions of the intestine, as in hysteria.

The normal color of feces is dark brown, due to the changed bile pigment, urobilin. The color in nurslings is a light to a golden yellow. Diet affects the color perceptibly. In a milk diet, or one rich in fats, the stools are light; a great indulgence of meat gives to the dejecta a brownish-black appearance, due to hematin and iron sulphide. Red wine and black cherries produce dark stools. Drugs also affect the color. Calomel produces a green tinge, due to the reunion of mercaptan with mercury; iron and bismuth preparations produce a black stool, because of the combination with sulphides. The stool becomes darker a short time after evacuation, due to the oxidising influence of the oxygen in the atmosphere. Pathologically the stool becomes darker in constipation and in intestinal hemorrhage. In acholics, where no bile enters the intestinal canal, either because of duodenal enteritis or closure of the biliary passages, the dejecta are very light. They are light not only because of the absence of bile pigments, but also because of the presence of undigested and unabsorbed fats.

The disagreeable odor of the dejecta is due to skatol and indol, aromatic substances, resulting from the putrefaction of the proteids. Because of the more pronounced putrefactive changes in enteric catarrh the odor becomes very foul, due to the presence of sulphuretted hydrogen and water gas. According to Sahli,[2] the stool is normally weakly acid and pathologically it may be strongly acid or alkaline, dependent upon the kind of putrefactive process in the intestinal canal.

Microscopically, in a normal stool, many bacteria are found, and of these I will speak later. Beside the bacteria the food remnants or undigested food stuffs are recognisable. Normally, the undigestible portions of the food again appear. To this class belong all plant cells of fruits and vegetables rich in cellulose. Starch is detected by its well-known picture. The finding of many starch cells in the stool of adults is pathological and indicative of a diarrhea from the small intestine, or hyperacidity of the gastric juice. After partaking of meat, every stool contains muscle fibers. They are easily to be recognised because of the well-preserved striations and are usually stained with the bile pigment. As their digestion becomes more advanced the transverse striations become less prominent and the corners of the particles become more and more rounded. Muscle fibers in the stool become increased in diseases of high temperature and disturbances of physiological chemistry. Large connective tissue bands and pieces of elastic tissue are also remnants of animal nourishment.

Fat may be found in the stool after a mixed diet, and its quantity is dependent upon the amount of fat ingested. It may occur saponified, *i. e.*, united with basic salts, neutral, or as fatty acids. It is rarely found in globules, except in a rich milk diet, but it is more frequently seen as needle-like crystals. The fatty needles represent the fat difficult of emulsification and, therefore, of resorption. The stool can abnormally be rich in fats (steatarrhea). This phenomenon is present in diseases of the pancreas, such as carcinoma, fatty necrosis and in acholie. The stool is also rich in fats where there is no resorption of fat possible, because of atrophy of the intestinal mucosa, as in tuberculosis or amyloid degeneration, or where there is a caseation of the mesenteric lymph nodes, or a chronic tubercular peritonitis.

The following salts are found in the feces : (1) triple phosphates, which are usually bile-stained ; (2) calcium oxalate crystals, especially after a vegetable diet ; (3) cholestrin, which is seldom found in the characteristic form. Squamous epithelial cells are seldom seen, but cylindrical cells with nuclei are found. They are most frequently passed in a thin diarrheal stool.

Having thus far referred to substances which are found in the normal feces, I would call your attention to such matter which, if found in appreciable quantities, points toward disease. Charcot crystals, pointed and spindle-form, are sometimes found. According to Leichtenstern, Nothnagel and Boas, they are pathognomonic for intestinal parasites. I recall a case which came to I. Boas, in Berlin, for consultation. An American had been sent to Carlsbad by a New York physician. Boas, in all his cases, systematically examines not only the stomach contents but also the feces. In the examination of the stool, Boas discovered the presence of Charcot crystals, and concluded to put the patient on anthelmintic treatment. The parasite was passed intact. The patient recovered and did not go to Carlsbad.

Mucus can always be determined in the normal adult stool by its chemical reaction. If to that portion of the stool containing mucus, acetic acid be added, it will become opaque and translucent. If present, so that it is easily to be recognised, macroscopically, it denotes a catarrhal condition of the intestines. (1) Mucus may be found surrounding the stool ; (2) it may be equally mixed or incorporated in the fecal mass ; (3) or it may be passed by itself in large fibrous bands or smaller particles. Mucus surrounding the stool externally has its origin from the large intestine ; mucus equally distributed in the stool denotes a catarrhal condition of the small intestine ; mucus passed by itself in long bands and in the intervals of a normal stool, especially if accompanied by colicky pain, is pathognomonic of a disease described by Nothnagel and called by him enteritis membranacea or colica mucosa.

In discussing bloody stools it would be well to first call attention to some of the disease processes of which we must think, should it be present : (1) Toxic inflammations and strong drastic cathartics. (2) Traumatisms. (3) New growths, as carcinomata and polypi. (4) Circulatory diseases : (*a*) embolism of the superior mesenteric artery, a condition coming on in disease of the heart and general atheroma. It occurs in the feeble and the aged and is accompanied by all the symptoms of peritonitis, great pain, tense, rapid pulse, meteorism and the passage of blood ; (*b*) general venous hyperemia ; (*c*) congestion of the portal system ; (*d*) small aneurisms of intestinal vessels ; (*e*) venous varicosities, especially in the small intestine ; (*f*) amyloid degeneration of the vessel walls. (5) Diseases of the blood : (*a*) pernicious anemia and leukemia ; (*b*) grave intermittent fever ; (*c*) hemoglobinuria. (6) Diseases of the stomach : (*a*) carcinoma ; (*b*) ulcus ventriculi ; (*c*) acute gastritis. (7) Diseases of the intestine : (*a*) acute enteritis ; (*b*) amebic dysentery ; (*c*) ulcerations from typhoid fever ; (*d*) carcinoma ; (*e*) duodenal ulceration ; (*f*) incarcerated hernia ; (*g*) anchylostomum duodenalis ; (*h*) fissure and hemorrhoids. (8) Constitutional diseases : (*a*) scorbutus ; (*b*) purpura hemorrhagica; (*c*) phthisis ; (*d*) diabetes mellitus.[3] (9) Infectious diseases : (*a*) typhoid fever ; (*b*) yellow fever.

Blood is most frequently encountered in typhoid fever, amebic dysentery, carcinoma of the colon and hemorrhoids. The bloody contents of the stool vary ; blood may be seen microscopically and sometimes macroscopically, because of the brown or red color which it imparts to the stool. In the latter case the origin of the hemorrhage may be determined from the color and arrangement of the blood mixture. A solid, well-formed stool, covered externally, points to hemorrhage at the lowest point of the intestine, and indicates hemorrhoids, but a homogenous, bloody-tinged and hardened fecal mass indicates the seat of hemorrhage in the upper part of the intestinal tract, to the small intestine. The etiological nature of the hemorrhage may point to its localisation. A hemorrhage during typhoid fever would be seated in the lowest portion of the ileum; during amebic dysentery, in the large intestine; and after a burn, in the duodenum.

The blood, in view of its passage through the digestive tract, undergoes, because of the digestive, fermentative and putrefactive processes, a series of changes. The important derivatives of the hemoglobin arising are methemoglobin, hematin, hematoidin and urobilin. Urobilin is normally found in the stools, a derivative of the bile pigment, and Kirk[4] says that hematoidin is identical with bilirubin, a normal bile pigment. The detection of these derivatives is best ascertained by use of the spectroscope, but this means is often, however, very difficult, for the blood corpuscles are quickly destroyed in the stomach and intestinal tract, and the characteristic resorption lines of methemoglobin—hematin—may fail and be replaced by indistinct lines of allied derivatives.

There may be occasion to differentiate between certain drugs and a hemorrhage. So far as iron preparations are concerned, the color alone will decide, but for the bismuth preparations the microscope should be used and the hematin and bismuth crystals can then be easily differentiated.

From the variety of the pus mixture in the dejecta, we draw the same conclusions as to the origin as those at which we arrived in the discussion of blood in the stool. When pus can be detected only microscopically it denotes catarrhal or new-growth changes in the mucosa of the intestines. If pus be evacuated alone it points to a perforating abscess, and if mixed with blood or mucus it denotes either dysentery or ulcerating carcinoma in the region of the rectum or lower colon. The differential diagnosis of the latter two diseases will, of course, easily be made by digital rectal examination.

The detection of pus cells, microscopically, is often difficult, because here also, as with the blood, the digestive processes exercise a destructive action, and it is arduous to differentiate them from animal cells in the food stuffs or leucocytes detached from the mucosa.

In chololithiasis, gall stones may intermittently, after a preceding colic or not, be emptied into the stools, and in order to find them the stool must be filtered through a narrow-meshed sieve. Their physical appearance and chemical composition is familiar to you. It is important to know whether the gall-stones are faceted or round. Faceted stones are always multiple and occur in the gall-bladder, whereas round stones are found multiple or solitary and may originate either in the gall-bladder or biliary passages.

The subject of animal parasites in the feces is of such vast importance that but little justice could be done to it in this paper. Before dismissing the subject I would emphasise the significance of examining microscopically the stool for the eggs and extremities of parasites, and what a hint the finding of Charcot crystals should give to you. In vertigo,[5] convulsions, epileptiform attacks, psychical disturbances and in grave and pernicious anemias do not forget to look for the animal parasites within the intestinal tract. The prognosis in typhoid fever becomes grave if at any time ascarides lumbricoides are evacuated in the stool. The "exitus" is preceded by a fatal hemorrhage or a perforative peritonitis. I saw two such cases end in this manner—one while house physician at the Rochester city hospital, the other in Leyden's ward, at Berlin.

Normally and pathologically, the digestive tract contains many microörganisms. They have a physiological rôle in that they are productive of fermentative and putrefactive changes, by which they influence the body economy. In 1683, Leeuwenhoek, of Delft, Holland, called attention to microörganisms in the mouth and feces, and because of the regularity of contour he called them animalcules. Frierichs, 130 years later, described fungi and sarcinæ as inhabitants of the intestinal canal, but did not attach any significance to them. Billroth examined the meconium and found it free from colonies, but in the first golden-yellow stool of the new-born he noticed many bacteria, which he called coco-bacteria. Billroth showed that these so-called vegetations increased in number from the stomach downward and that in the large intestine there were many microörganisms, some showing motility, others not.

With the discovery of plate culture by Koch[6] the closer study of bacteria of feces obtained a great impetus. In 1883 it was begun by him by the isolation of the cholera vibrio in the stools of cholera cadavers.[7] Escherich, in 1885,[8] discovered the bacillus coli communis and the bacillus lactis ærogenes. He made a classical study of the bacteria of the feces and draws the same conclusions, proven by the growth and isolation of colonies on plate media, which Billroth vaguely described beforehand : (1) that the first golden-yellow stool contains bacteria ; (2) that the number and variety increase from the stomach downward.

Bacteria are introduced into the intestine by the mouth, (air, food, sputum, nasal secretions,) by the anus and by the blood. According to Schild,[9] the early infection of the intestinal tract arises from the anus and occurs from four to seventeen hours after birth. Late infection originates either by the mouth, or anus, or both. The bacteria come partly from the air, partly from the bathing water and seldom from the clothing or vagina of the mother. Escherich and Schild both found bacilli, cocci and fungi in the stool as soon as milk was taken. According to Sucksdorf,[10] the amount of bacteria are diminished by sterilising the food stuffs, but the gastric secretion has little or no effect on their growth. The bacteria, when once introduced into the intestinal canal, find all the requirements for their multiplication : (1) a good medium for their growth ; the proteids are changed to soluble peptones, the fats are emulsified and the starches are transformed into glucose : (2) heat, *i. e.*, the bodily temperature ; (3) moisture.

As before stated, the bacteria play a physiological role. They are the organised ferments and are to be differentiated from trypsin, ptyalin, and the like, which are unorganised ferments. Hoppe-Seyler[11] illustrates their action by several examples :

Lactic fermentation, bacterium lacticum —Cohn

Milk sugar. Grape Sugar. Lactic Acid.

$$C_{12}H_{22}O_{11}+H_2O = 2\,(C_6H_{12}O_6) = 4\,(H_3C_6O_3.)$$

Butyric fermentation, by bacillus subtilis.

Lactic acid. Butyric acid. Carbonic acid.

$$2\,(C_3H_6O_3)+2\,(H_2O)=C_4H_8O_2 + 2\,(H_2CO_3) + H_4.$$

Putrefactive processes, the ferments of which have not been isolated.

$Albumen + N(H_2O) =$

Globulin.

Peptone.

Leucin.

Tyrosin.

Xanthin.

Indol, phenol, scatol.

Fatty acids.

Carbonic acid.

Ammonia.

Sulphuretted hydrogen.

Mannaberg[12] describes fourteen varieties of bacilli, nine of cocci and four of fungi in the feces. Eisenberg[13] enumerates some twenty-four varieties of microörganisms, of which ten are non-pathogenic and the remainder are pathogenic. Beside their physiological function, a majority of the bacteria are, under certain conditions, pathological and a few are specific in certain well-known diseases. So long as the bacillus coli communis remains in the large intestine it shows no virulence, but when once it wanders from the normal seat of its habitation it produces septic symptoms. It is the productive factor in perityphlitis and appendicitis. It produces sulphrenic abscesses, cholangitis, cholecystitis, peritonitis, pericarditis, meningitis, pyelitis, pyelonephritis, tonsillitis and cystitis. Schnitzler[14] claims that the bacillus coli communis always produces an alkaline cystitis in contradistinction to the gonococcus of Neisser, which causes an acid cystitis. A bacillus hard to differentiate from the bacillus coli communis and specific for typhoid fever is the bacillus of typhoid fever (Eberth). It is found in the feces of typhoid patients and is a constant factor in the production of the disease.

As before mentioned, Koch found in cholera the vibrio cholera, and here it may be added that cholera Asiatica cannot be diagnosticated unless this organism can be found and isolated in the stool of a suspected patient. The cholera vibrio is hard to differentiate from a great variety of vibrio found in water and from the Prior-Finkler vibrio of cholera nostra. In phthisical patients the sputum is often swallowed, and because of the absence of hydrochloric acid, usually noted in such cases, the tubercle bacillus enters the intestinal tract, having lost none of its virulence, and a tubercular enteritis or peritonitis may follow. The bacilli in such a stool may be found by spreading some of the feces on a cover glass, fixing and staining either by the Koch-Ehrlich or Gabbet method.

Lesage[15] describes a bacillus of green diarrhea, and Salus[16] a case of green coloring of the stool by the bacillus pyocyaneus. Various staphylococci and streptococci are also present in the stool, and they have much to do with terminal infections. Lastly, there were demonstrated to me, in the stool as well as in the stomach contents, two organisms upon which Boas[17] attached diagnostic importance—namely, the sarcinæ ventriculi, a form of cocci which multiply in all directions and found in gastrectasis, and the so-called faden bacilli in gastric carcinoma.

In connection with the examination of feces, the examination of urine should always be undertaken. It has been shown that aromatic substances, such as cresol and skatol, indol and phenol, are produced by anerobic bacteria on proteids and albuminoid foods. Bauman, in 1880, showed that these aromatic substances were eliminated in the urine in the form of ethereal sulphates. When found in the urine they are, therefore, an index whether the putrefactive processes in the intestinal tracts are or are not excessive. Indican is derived from indol. Salkowski has found it in the urine in perityphlitis and appendicitis ; Braeger in chronic anemia and cachexia ; Jaffe in malignant disease of the intestine ; Senator in chronic peritonitis and gastric carcinoma. Hester and Smith found it increased during attacks of epilepsy. In stricture of the small intestine, indican becomes very much increased in the urine, whereas in closure of the large intestine it is seldom present. It is, therefore, of great use clinically in determining whether the seat of stricture is in the small or large intestine.

There still remains much to be learned in clinical examination of feces. It is a field rich for scientific work and experimentation, and one in which brilliant results are yet to be obtained.

REFERENCES.

1. Prof. Dr. H. Nothnagel Die Erkrankung des Darms und des Peritoneum. 1895. S. 8.
2. Sahli. Untersuchungsmethoden. Leipzig und Wien. S. 347.
3. Loeb. *Deutsche Med. Zeitung.* 1894.
4. Kirk's Handbook of Physiology, p. 88.
5. Lehrbuch der Nervenkrankheiten. Von Prof. Dr H. Oppenheim. 1894. P. 728.
6. Koch, Robt. Mitth. aus d. Kais. Ges. Amte. Bd. I. 1881. P. 27, Zeile 25–29, und p. 36, Zeile 11–14.
7. Dr. Carl Gunther. Einführung in das Studium der Bakteriologie. Dritte Auflage. P. 267.
8. Escherich. *Fortschr. der Medizin* 18-8. No 15.
9. Schild. *Zeitschrift für Hygiene und Infectionskrankheiten.* XIX. 1, 1895.
10. W. Sucksdarf. Das Quantativen Vorkommen von Spaltpilzen im Menschlichen Darm Canal. *Archiv. f. Hygiene*, Bd. IV. 1886.
11. Geo. R. Fowler. Digestion and Diet ; their Relation to Disease. *Brooklyn Medical Journal*, Vol. I., No. 6, June, 1888.
12. Julius Mannaberg Die Bakterien des Darms in die Erkrankung des Darms. Nothnagel.
13. Eisenberg. Bakteriolog. Diagnostik. XXIX.
14. Jul. Schnitzler. Zur Etiologie der Cystitis. Wien. 1892.
15 Lesage. In bacille de la diarrheé verte des enfants, du premier age. *Arch. de Physiologie.* 1888. No. 1.
16. Salus *Prager Med. Wochenschrift.* 1894. 33.
17. I. Boas. Diagnostik und Therapie der Magenkrankheiten. II. Theil. P. 100 and p. 182.
18. Herter and Smith. Observations on Excessive Intestinal Putrefaction. *New York Medical Journal*, June and July, 1895.

400 FRANKLIN STREET.

